# Managed entry agreements

**DOI:** 10.1186/1750-1172-9-S1-O27

**Published:** 2014-11-11

**Authors:** Luisa AA Muscolo, Claudia Bernardini, Paolo D Siviero, Simona Montilla, Luca Pani

**Affiliations:** 1 Italian Medicines Agency (AIFA) Rome, Italy

## Background

New scientific progress in the “precision medicines” direction and a better diagnosis of rare diseases have led Regulators and Payers to focus their vigilance towards the real life setting. Although Regulators and Payers are taking into consideration patient pressures for rapid access to treatment, nevertheless they must balance the difficulties of taking significant decision coping with uncertainties when deciding on pricing and reimbursement processes.

## Materials and methods

A large number of mechanisms (Managed Entry Agreements) have been developed to limit the reimbursement of medicines to those subpopulations that are most likely to benefit from treatment. MEA are playing a key role in bridging the possible gap by the management of uncertainty in knowledge relating to pricing and reimbursement of new medicines.

Furthermore, monitoring registries represent one of the most advanced experience through all developed MEA tools. The aim of these registers is to define patient eligibility to a treatment by ensuring the proper use of the medicinal product according to the approved indications and concerning decision taken on reimbursement.

According to the variety of mechanisms and the way they are structured, MEAs offer a wide flexibility able to deal with different types of uncertainties at the same time through the monitoring and combination of financial and performance-based agreements (e.g. budget impact, weakness in clinical evidence, etc).

A taxonomy has been developed within the European project “Capacity building on managed entry agreements for innovative medicines” in order to enable the provision of a solid guide in the process of identifying the most appropriate scheme to be adopted in each specific situation.

Regarding the objective of the MoCA project to identify the pathway, that may facilitate the access of OMP to the market, it was very hard to find agreements among all participants on how to facilitate the access of orphan drugs in the real life setting, given the high cost and the lack of evidence of these products.

## Results

Hence the use of MEAs representing a significant tool for the management of a sustainable pharmaceutical expenditure and also for the generation of further clinical evidences by registries’ adoption, they can be considered the key element to overcome this concern.

## Conclusions

Through the sharing at European level of a standardized collection of evidences obtained with these instruments, it would be possible to collect a large and robust amount of clinical evidences to be used for clinical research development.

